# Recent Advances in the Siderophore Biology of *Shewanella*

**DOI:** 10.3389/fmicb.2022.823758

**Published:** 2022-02-17

**Authors:** Lulu Liu, Wei Wang, Shihua Wu, Haichun Gao

**Affiliations:** Institute of Microbiology, College of Life Sciences, Zhejiang University, Hangzhou, China

**Keywords:** siderophore, siderophore biosynthesis, iron uptake, *Shewanella*, regulation

## Abstract

Despite the abundance of iron in nature, iron acquisition is a challenge for life in general because the element mostly exists in the extremely insoluble ferric (Fe^3+^) form in oxic environments. To overcome this, microbes have evolved multiple iron uptake strategies, a common one of which is through the secretion of siderophores, which are iron-chelating metabolites generated endogenously. Siderophore-mediated iron transport, a standby when default iron transport routes are abolished under iron rich conditions, is essential under iron starvation conditions. While there has been a wealth of knowledge about the molecular basis of siderophore synthesis, uptake and regulation in model bacteria, we still know surprisingly little about siderophore biology in diverse environmental microbes. *Shewanella* represent a group of γ-proteobacteria capable of respiring a variety of organic and inorganic substrates, including iron ores. This respiratory process relies on a large number of iron proteins, *c*-type cytochromes in particular. Thus, iron plays an essential and special role in physiology of *Shewanella*. In addition, these bacteria use a single siderophore biosynthetic system to produce an array of macrocyclic dihydroxamate siderophores, some of which show particular biological activities. In this review, we first outline current understanding of siderophore synthesis, uptake and regulation in model bacteria, and subsequently discuss the siderophore biology in *Shewanella*.

## Introduction

Iron is one of the most abundant metal elements on the Earth and displays a wide range of oxidation-reduction potential, a chemical property largely resulting from the transition between two stable valences, the ferrous (Fe^2+^) and ferric (Fe^3+^) forms (Andrews and Schmidt, 2007). For nearly all living organisms, iron on one hand is essential because iron-dependent proteins are employed to perform a myriad of functions in diverse biological processes, such as electron transport, metabolism, peroxide reduction, amino acids and nucleoside synthesis, DNA synthesis, photosynthesis, and gene expression (Andrews et al., 2003; Wandersman and Delepelaire, 2004; Payne et al., 2015). To act as protein cofactors, iron molecules may require an assembly process to form active complexes, such as iron-sulfur clusters and heme, or interact with apoproteins transiently as mono- and bi-nuclear iron centers (Solomon et al., 2000; Py and Barras, 2010; Rokob et al., 2016). On the other hand, the overloaded iron could be extremely toxic to cells by catalyzing the formation of potentially lethal reactive oxygen species (ROS) (Imlay, 2013). As a result, iron homeostasis in living cells has to be carefully maintained through coordinated expression of proteins involved in iron uptake, storage, and consumption, which are tightly regulated at both transcriptional and post-transcriptional levels (Andrews et al., 2013).

In bacteria, a variety of iron-uptake systems have been identified and characterized. Among them, Feo, a transporter functioning to specifically acquire Fe^2+^ from environments, is the most critical in supporting normal metabolism and growth in bacteria (Lau et al., 2016). Most bacterial Feo systems are composed of two subunits, FeoA and FeoB, but variations exist, including a three-subunit (FeoA, FeoB, and FeoC) Feo system found in *Escherichia coli* and some other γ-proteobacterial species, a single fused FeoA/FeoB protein, and an isolated FeoB whose FeoA remains to be identified (Lau et al., 2016). In well characterized two-subunit Feo systems, FeoB functions as an Fe^2+^ permease with a cytosolic N-terminal G-protein domain and a C-terminal integral inner-membrane domain containing two ‘Gate’ motifs whereas FeoA is a small-molecule hydrophilic protein required for Feo function by promoting the formation of the Feo complex (Lau et al., 2013; Seyedmohammad et al., 2016).

**FIGURE 1:**
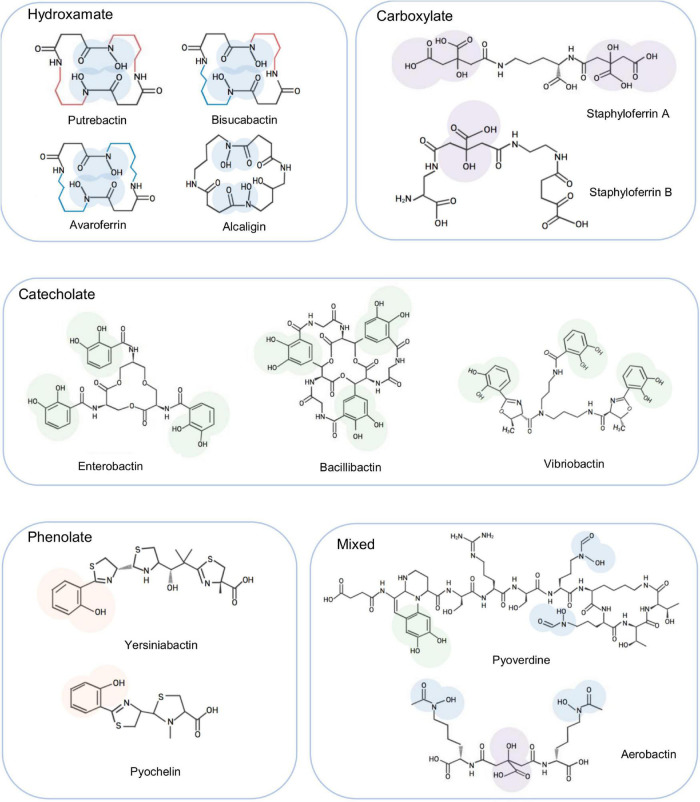
Representative siderophores synthesized by bacteria. To show the characteristics of each type, the iron binding moieties of these siderophores are highlighted. These siderophores are also mentioned in the text as examples to facilitate illustration of siderophore biosynthesis and transport.

Despite the abundance of iron and the effectiveness of Feo for Fe^2+^ uptake, iron acquisition still represents a major challenge for many microorganisms because iron forms insoluble ferric hydroxides in the presence of oxygen, reducing the level of soluble Fe^2+^ below the threshold of Feo (de Carvalho et al., 2011; Niessen and Soppa, 2020). To overcome this, an impressive and widespread strategy, the siderophore-dependent iron uptake, designed to solubilize and capture iron from aerobic environments, has evolved (Hoegy et al., 2005). Siderophores, produced by both prokaryotes and eukaryotes, are low-molecular-weight components (500–1,500 daltons) with a high affinity for insoluble Fe^3+^ (de Carvalho et al., 2011). Siderophores are commonly classified according to their iron-binding moieties: catecholate, hydroxamate, phenolate, carboxylate, and mixed-type, which contains more than one of the aforementioned moieties (Figure 1). Biosynthesis of siderophores is catalyzed by two types of enzymatic machines, non-ribosomal peptide synthetase (NRPS) modular multienzymes and NRPS-independent (NIS) enzymes (Barry and Challis, 2009). NRPS enzymes, widely recognized for their selectivity in assembling specific peptide products, use the repeating groups of catalytic domains to install one monomer into the growing peptide (Bloudoff and Schmeing, 2017). Siderophores synthesized by NIS enzymes, mostly hydroxamates, are assembled through the oligomerization and macrocyclization of γ-aminocarboxylic acid substrates via multiple rounds of amide bond formation (Barry and Challis, 2009; Codd et al., 2018). In addition, multiple NIS synthetases could work together to participate in a unique hybrid NIS-NRPS pathway for biosynthesis of siderophores (Lee et al., 2007; Oves-Costales et al., 2007).

**FIGURE 2:**
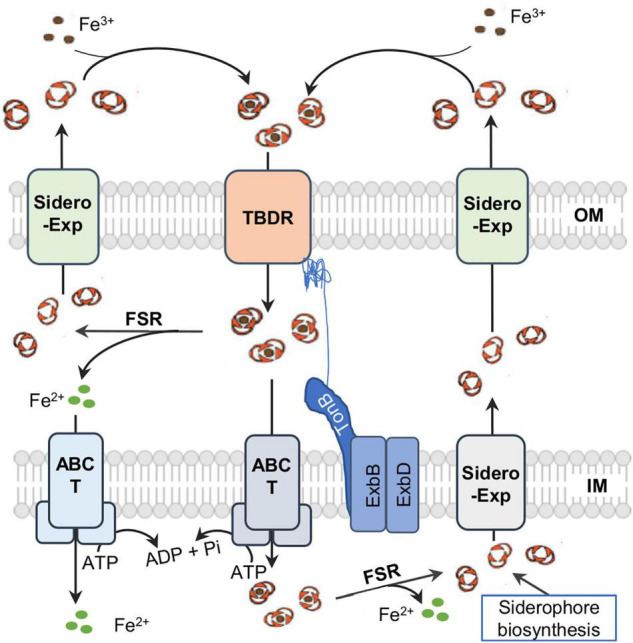
A simplified view of siderophore-meidated iron transport in Gram-negative bacteria. Siderophores are synthesized in the cytoplasm and exported by diverse exporters (Exp) on the IM (inner membrane) and OM (outer membrane). Ferrisiderophores are imported into the periplasm by TonB-dependent receptor (TBDR) on the OM. In most cases, ferrisiderophores are subsequently transported by transporters, mostly ABC transporters (T), into the cytoplasm, where reduction takes place by a specific ferrisiderophore reductase (FSR) to release iron in the ferrous form. In some rare cases, ferrisiderophores in the periplasm are directly reduced to release ferrous iron and siderophore molecules, which then are transported across the IM by a specific ABC transporter and across the OM by siderophore exporter for reuse respectively.

In Gram-negative bacteria, once produced, siderophores are exported by specific transporters into the surroundings, where they bind Fe^3+^ to form ferrisiderophore complexes (Figure 2). The complexes are recognized and trafficked across the outer-membrane (OM) by a TonB-dependent receptor (TBDR) depending on the energy transduced by the TonB-ExbB-ExbD system located in the IM, and subsequently translocated across the inner-membrane (IM) by the activity of ATP-binding cassette (ABC) transporters or permeases into the cytoplasm (Schalk and Guillon, 2013). In the cytosol, Fe^3+^ in the ferrisiderophore complex is reduced by ferrisiderophore reductase (FSR) to Fe^2+^ and released from the complex (Crosa and Walsh, 2002; Noinaj et al., 2010). In some cases, iron dissociation from ferrisiderophore complexs occurs in the periplasm either by modification of the siderophore scaffold or by reduction, and the resultant free siderophores can be exported directly for reuse (Schalk and Guillon, 2013).

Given that siderophores play an important role in bacterial physiology, their biosynthesis, transport, and recycling are under tight regulation as a critical means to maintain intracellular iron homeostasis. Two main iron-responsive regulators, the ferric uptake regulator (Fur) and small RNA RyhB that are widespread in many bacteria, are particularly important (Andrews et al., 2013; Chareyre and Mandin, 2018). In *E. coli*, Fur has been initially identified to be an iron-dependent repressor upon binding to Fe^2+^ for transcription of many genes involved in iron homeostasis by interacting with the specific motif in DNA promoter regions (Fillat, 2014), while RyhB serves as an antagonist factor against Fur regulation by mainly repressing the expression of iron proteins (Massé et al., 2005).

Shewanellaceae, a family within the order Alteromonadales belonging to the class γ-proteobacteria, consist of a sole genus *Shewanella* (Satomi, 2014). *Shewanella* comprise a group of facultative dissimilatory metal-reducing bacteria that are common in water and sedimentary environments that are chemically stratified and renowned for their respiratory versatility (Fredrickson et al., 2008). Such trait has been extensively investigated and exploited for biotransformation of solid metal oxides from wastewaters and electricity generation in microbial fuel cells (Logan et al., 2006; Fredrickson et al., 2008). In addition, some *Shewanella* species are well-known spoilers of food products and notorious fish pathogens (Janda and Abbott, 2014; Lemaire et al., 2020). The respiratory versatility is largely attributable to a large repertoire of iron-containing proteins, iron-sulfur proteins and hemoproteins in particular, a feature requiring relatively high iron contents compared to model bacterial paradigm *E. coli* (Daly et al., 2004; Fu et al., 2018; Liu et al., 2020). Because of this, *Shewanella* spp., as in the extensively studied representative *Shewanella oneidensis*, have evolved novel physiological characteristics to maintain iron homeostasis.

The aim of this review is to elucidate the siderophore-dependent aspects by summarizing the recent advances in siderophore biosynthesis, secretion, uptake, regulation, and its physiological impacts on *Shewanella*. In each aspect, we outline current understanding derived from relevant bacteria that helps pinpoint the characteristics of *Shewanella*. Additionally, we also discuss the impacts of the endogenous siderophores on biosynthesis of cytochromes *c*, on physiological processes associated with extracellular electron transfer, and on biofilm formation in *Shewanella*.

## Siderophores and Siderophore Biosynthesis

Siderophores were first detected in *Shewanella* species over two decades ago and in *Shewanella putrefaciens* they were soon identified to be putrebactin, a cyclic homodimer of succinyl-(*N*-hydroxyputrescine) produced from a single substrate, putrescine (Gram, 1994; Ledyard and Butler, 1997; Figure 1). In addition to putrebactin, bisucaberin and avaroferrin have been identified later from *Shewanella algae* B516 (Böttcher and Clardy, 2014; Figure 1). While bisucaberin, the same as putrebactin, is a homodimeric product of two molecules of cadaverine, avaroferrin is generated from the combination of two substrates putrescine and cadaverine (Kameyama et al., 1987; Rütschlin et al., 2017). As cyclic dihydroxamates with flat structure, all of these three siderophores require significant conformational rearrangement to coordinate iron (Watrous et al., 2013).

**FIGURE 3:**
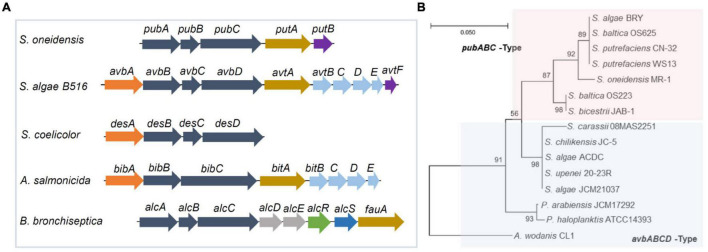
Gene clusters for biosynthesis of cyclic dihydroxamate siderophores in bacteria. **(A)** The synthetic gene cluster. Genes in dark blue, NIP systems; in orange, decarboxylases; in brown, TBDRs; in purple, siderophore reductases; in light blue, siderophore ABC transporters for entering the cytoplasm; in sky blue, siderophore exporter; in green, transcriptional regulator; in red, MFS-family siderophore exporter; in gray, functionally unknown. **(B)** Phylogenetic analysis of representative *Shewanella* having the Pub and Avb systems. Shown was a maximum likelihood tree generated using 16s rRNA DNA sequences with 1,000 bootstrap repetitions. Included are *Shewanella* strains carring a *pub* cluster (pink area) as well as *Shewanella* strains and three other bacterial strains carrying an *avb* cluster (blue area). S, *Shewanella*; P, *Pseudoalteromonas*; A, *Aliivibrio*.

The biosynthesis of putrebactin, avaroferrin, and bisucaberin is catalyzed by a NIS system, dubbed PubABC in *S. putrefaciens* and AvbBCD in *S. algae* B516, which consists of three subunits functioning as N-hydroxylase (PubA/AvbB), acylase (PubB/AvbC), and lucC-like synthetase (PubC/AvbD) (Kadi et al., 2008a; Böttcher and Clardy, 2014). Bacterial homologies of the PubABC/AvbBCD systems include DesBCD of *S. coelicolor*, MbsBCD of an unknown bacterium and BibBC of *Vibrio salmonicida* in which BibC is a fusion protein possessing activities of both PubB and PubC, and AlcABC of *Bordetella bronchiseptica*, which are responsible for the biosynthesis of desferrioxamine E, bisucaberin, and alcaligin respectively (Figure 3A; Barona-Gómez et al., 2004; Kadi et al., 2007, 2008a,b; Fujita et al., 2012; Li B. et al., 2016; Codd et al., 2018). Initially, these systems were proposed to function in a substrate-specific manner because only one single siderophore was identified from bacteria hosting any of them (Ledyard and Butler, 1997; Kadi et al., 2008a). However, recent studies revealed that AvbBCD can produce not only putrebactin, bisucaberin and avaroferrin, but also a variety of homodimeric and heterodimeric combinatorial products ranging from 18- to 28-membered rings provided that the required substrates are supplemented (Böttcher and Clardy, 2014; Rütschlin et al., 2018). Hence, it is conceivable that all of these synthesizing systems may be able to generate an array of cyclic hydroxamate molecules and that the substrate pool of precursor molecules dictates the ratios of the final products in the cell although substrate preference may also be a factor (Rütschlin et al., 2018).

Consistent with this notion, two siderophores other than putrebactin have been detected in PubABC-carrying *S. putrefaciens* before but neither was identified successfully due to the low quantity (Soe et al., 2012). By using a precursor-directed biosynthesis approach, putrebactin, bisucaberin, and avaroferrin were simultaneously produced by *S. putrefaciens* with precursor supplementation and confirmed (Soe and Codd, 2014; Soe et al., 2016). The difference in the abundance of bisucaberin and avaroferrin between *S. algae* B516 and *S. putrefaciens* has been attributed to AvbA of the former, which is missing in the latter (Böttcher and Clardy, 2014; Rütschlin et al., 2017). AvbA is a lysine decarboxylase (LDC) responsible for biosynthesis of cadaverine, the essential substrate of bisucaberin and avaroferrin (Böttcher and Clardy, 2014). In *Shewanella* whose genome sequences are available by now, most species possess a PubABC-type siderophore synthetic system whereas the remaining are equipped with AvbBCD (Wang et al., 2020). The gene clusters for these two systems differ significantly (Figure 3A). For the PubABC system, operon *putAB* encoding a TBDR (PutA) and a FSR (PutB) follows the *pubABC* operon immediately (Liu et al., 2018). Both PutA and PutB are essential for siderophore-mediated iron uptake in *S. oneidensis* (Liu et al., 2018). On the contrary, there are 5 more genes in the *avb* cluster of *S. algae* B516 (Böttcher and Clardy, 2014). In addition to *avbA* mentioned above, four additional genes separate *avtF* (*putB*) from *avtA* (*putA*). These four genes encode an IM ABC transporter, presumably responsible for the uptake of the ferrisiderophore complex across the IM (Rütschlin et al., 2017; Wang et al., 2020). Intriguingly, a phylogenetic analysis reveals that *Shewanella* species with the same gene organization are clustered together, even for different strains of the same species (Wang et al., 2020; Figure 3B). This strong relatedness implies that these two gene clusters are likely formed by gene shuffling and periodic selection within the genus rather than horizontal gene transfer (Wang et al., 2020).

Although the lack of LDC AvbA in the PubABC-carrying *Shewanella* readily explains the low quantity of bisucaberin and avaroferrin, there must be the enzymatic sources for cadaverine generation. Both putrescine and cadaverine are derived from the reactions catalyzed by a group of enzymes called basic amino acid decarboxylases (BAADs) (Carriellopez et al., 2018). In bacteria, it is common that multiple BAADs are present, including LDC, arginine decarboxylase (ADC), ornithine decarboxylase (ODC), and importantly a large portion of them are known to be promiscuous for substrates (Burrell et al., 2012; Tomita et al., 2014; Carriellopez et al., 2018). Consistently, there are several BAADs in *S. oneidensis*, including ADC SpeA, ODCs SpeC and SpeF, LDC SO_1550 (based on the genome annotation) and SO_1769, a homolog of *S. algae* B516 AvbA (BLASTp E-value, 4e-48) (Wang et al., 2020). The primary pathway of *S. oneidensis* for putrescine production is composed of ADC SpeA, agmatine deiminase (AguA), and *N*-carbamoylputrescine amidohydrolase (AguB) rather than ODC (Szumanski and Boyle, 1990; Wang et al., 2020). In cadaverine generation, surprisingly, neither annotated LDC SO_1550 nor the AvbA homolog SO_1769 appears to play a significant role (Wang et al., 2020). Instead, both SpeC and SpeF are involved (Wang et al., 2020). Although both enzymes are annotated as ODC and display ODC activity, SpeC has high LDC activity and its absence abolishes cadaverine production. It is therefore reasonable to propose that SpeC is crucially responsible for cadaverine biosynthesis in PubABC-carrying *Shewanella*.

## Siderophore Transport

### Exporter for Siderophore Releasing

Once generated, siderophores must be secreted to the outside of the cell through specific exporters (Figure 2). Exporters known to transport siderophores across the membranes in bacteria are diverse, belonging to many transporter superfamilies, particularly ABC superfamily, the major facilitator superfamily (MFS), and the resistance-nodulation-division (RND) superfamily (Alav et al., 2021). However, because of the general conservation among members of the same exporter family, the functional overlapping/redundancy of multiple siderophore exporters, and timely regulation, it is rather difficult to precisely identify the exporters by assessing siderophore transport defects and siderophore toxicity. Hence, the current understanding of bacterial proteins responsible for the process remains rather limited.

The ABC superfamily contains a large group of diverse transporters, characterized by a dimeric transmembrane porter section (permease) and a cytoplasmic ATPase section providing energy coupling for the transport process (Thomas and Tampé, 2020). Examples of ABC siderophore exporters include *Salmonella enterica* and *E. coli* MacAB systems for salmochelin and enterotoxin respectively, *Pseudomonas aeruginosa* PvdRT-OpmQ and MdtABC-OpmB for pyoverdine (Zhu et al., 1998; Farhana et al., 2008; Yamanaka et al., 2008; Hannauer et al., 2012; Bogomolnaya et al., 2020). The MFS superfamily is composed of single-polypeptide transporters, whose general architecture contains 2 bundles of 6 (or 7) transmembrane (TM) α-helices with a central symmetry (Ranaweera et al., 2015). Characterized MFS siderophore exporters include *E. coli* EntS for enterobactin, *Staphylococcus aureus* NorA, CsbX of *Azotobacter vinelandii* for catecholates and AlcS (synonyms, Bcr or OrfX) in *Bordetella pertussis* and *Bordetella bronchiseptica* for alcaligin (Furrer et al., 2002; Page et al., 2003; Brickman and Armstrong, 2005; Deng et al., 2012; Figure 3A). The RND superfamily transporters contain 12 (or 13/14) TM helices and two large external loops between helices 1 and 2 as well as 7 and 8 (Nikaido, 2018). Although RND efflux pumps mainly export heavy metals, various drugs, lipids, and pigments, siderophores can also be transported via a proton antiport mechanism (Miethke and Marahiel, 2007). Examples include *E. coli* AcrAB, AcrAD, and MdtABC for enterobactin, *Bacillus anthracis* ApeX for petrobactin, *Vibrio cholerae* VexGH for vibriobactin, as well as *Mycobacterium tuberculosis* MmpL4 and MmpL5 for both mycobactin and carboxymycobactin (Horiyama and Nishino, 2014; Hagan et al., 2017; Kunkle et al., 2017; Sandhu and Akhter, 2017).

In *S. algae* B516, an ABC transporter encoded by genes *avtBCDE* following the *avbABCD* operon exhibits considerable sequence homology to BitBCDE, the bisucaberin exporter in *V. salmonicida* (Figure 3A), and therefore is proposed as an exporter for avaroferrin (Böttcher and Clardy, 2014). However, the direct evidence to support this notion is yet available. In the *S. oneidensis* genome, the counterpart of *avtBCDE* in the siderophore synthesis cluster is not found, and neither is an operon encoding a BitBCDE homolog (Wang et al., 2020). To date, whether *S. oneidensis* alike lose *avtBCDE* or *S. algae* B516 obtains it by horizonal gene transfer during the evolution process remains elusive. Despite this, it is reasonable to propose that *avtBCDE*-less *Shewanella* are equipped with siderophore exporters given that dozens of multidrug efflux systems of all transporter superfamilies are encoded (Table 1).

**TABLE 1:** Putative siderophore exporters of *S. oneidensis*.

Type	Permease	Length (a.a.)	Operon	Annotation
RND	SO0520	1,075		heavy metal efflux pump, CzcA family
	SOA0153	1,045	A0155-3	heavy metal efflux pump, CzcA family
	SO0945(AcrB)	1,067	0946-5	AcrB/AcrD/AcrF family protein
	SO1882	1,031	1881-2	AcrB/AcrD/AcrF family protein
	SO1923(AcrB)	1,020	1925-4-3	AcrB/AcrD/AcrF family protein
	SO3103	1,087	3102-3	Thiophosphate efflux pump component
	SO3279	1,026	3278-9	AcrB/AcrD/AcrF family protein
	SO3484	1,046	3483-4	AcrB/AcrD/AcrF family protein
	SO3492(MexF)	1,047	3492-3	RND multidrug efflux transporter MexF
	SO4014	1,023	4014-5	AcrB/AcrD/AcrF family protein
	SO4328	1,029	4327-8	HlyD family secretion domain protein
	SO4598	1,045	4596-8	heavy metal efflux pump, CzcA family
	SO4692	1,044	4692-3	efflux RND transporter
ABC	SO0821(MacB)	656	0820-2	Macrolide export ATP-binding/permease protein
MFS	SO4555	402		drug resistance transporter, Bcr/CflA family protein
	SO2280	407		bicyclomycin resistance protein
	SO3485(EmrD3)	382		drug resistance transporter, Bcr/CflA family protein
	SO2389(EmrD)	414		multidrug resistance protein D
	SO2373	423		drug resistance transporter, Bcr/CflA family protein
	SO4021(MdtL)	396		transporter, putative

### TonB-Dependent Receptors for Ferrisiderophores to Enter the Periplasm

Owing to the presence of an OM layer and a periplasmic space, transport systems for uptake of ferrisiderophore complexes are more complicated in Gram-negative bacteria than in Gram-positive bacteria. Most ferrisiderophore complexes, which are unable to pass through porins on the OM because of their large size (over 500 daltons), depend on TonB-dependent receptors (TBDRs) for entering into the periplasm (Noinaj et al., 2010; Figure 2). TBDRs constitute a large group of integral OM proteins, featured by a unique ‘β-barrel’ structure and performing multiple cellular functions, such as nutrient uptake, protein secretion, and adhesion (Doyle and Bernstein, 2019). TBDRs exhibit extremely high substrate specificity, that is, each transports only a specific siderophore, or in some cases, a few structurally related siderophores (Mislin et al., 2006; Greenwald et al., 2009; Hoegy et al., 2009). By spending energy transduced by TonB-ExbB-ExbD located in the IM, TBDRs recognize and bind ferrisiderophore complexes with high affinity and internalize the compounds into the periplasmic space (Hider and Kong, 2010). Upon the contact of ferrisiderophore complexes with their cognate TBDR, the receptor undergoes a conformational change in the secondary structure, which is sensed by TonB (Stintzi et al., 2000). In recent years, a new type of TonB-independent siderophore receptors has been identified, such as LbtU and FupA/B in *Legionella pneumophila* and *Francisella tularensis*, respectively (Chatfield et al., 2011; Ramakrishnan and Sen, 2014). Although the role of *F. tularensis* FupA/B in ferrisiderophore uptake is still under debate (Siebert et al., 2020), it is fully established that *L. pneumophila* LbtU is essential for siderophore (legiobactin) utilization (Cianciotto, 2015). Importantly, while this explains how TonB-less bacteria take up iron via siderophores, it offers a possibility that the TonB-posistive counterparts may also exploit this approach in addition to TBDR-mediated iron transport.

In *Shewanella* as in most other Gram-negative bacteria, a large number of TBDRs are encoded; for instance, *S. oneidensis* contains SO_0798, SO_1156, SO_1482, SO_2970, PutA(SO_3033), SO_3914, SO_4422, SO_4516, IrgA(SO_4523) and SO_4743 (Qian et al., 2011; Dong et al., 2017; Liu et al., 2018). Among them, PutA is the only one required for iron uptake mediated by endogenous siderophores. Despite this, other TBDRs are without physiological significance; they, at least some, are responsible for uptake of iron via siderophores produced and released by other bacteria in the surroundings (xenosiderophores) (Cornelis and Dingemans, 2013; Wilson et al., 2016). In *S. oneidensis*, the removal of both PutA and ferrous iron uptake system Feo nearly abolishes iron uptake completely, leading to an extremely severe defect in growth (Liu et al., 2018). Since this defect can be substantially relieved with the supernatants of the *E. coli*, *Vibrio harveyi*, *B. subtilis*, and *S. aureus* spent cultures, it is clear that *S. oneidensis* can import iron with siderophores released from these bacteria. *E. coli* produces two siderophores, enterobactin and aerobactin, which belong to catecholate- and mixed-types of siderophores respectively (Wilson et al., 2016). IrgA of *S. oneidensis* is highly homologous to *E. coli* FepA and *P. aeruginosa* PfeA, which recognize and bind to ferric-enterobactin (Buchanan et al., 1999; Gasser et al., 2016). Hence, it is very likely that IrgA is the TBDR for enterobactin in *S. oneidensis*. The TBDRs of *S. oneidensis* for siderophores produced by *B. subtilis* and *S. aureus* are yet unknown as multiple siderophores are produced by these bacteria (Drechsel et al., 1993; Ghssein et al., 2016). Moreover, some TBDRs may have unconventional activities; for example, SO_2970 has been implicated in iron reduction (Qian et al., 2011). It is worth mentioning that TBDRs may evolve rapidly to alter their substrate specificities because spontaneous single missense mutations in TBDR genes of *Bradyrhizobium japonicum* are found to be sufficient to confer on cells the ability to use synthetic or natural siderophores (Chatterjee and O’Brian, 2018). By this token, capacities of *Shewanella* species in utilizing xenosiderophores may vary significantly due to a large repertoire of TBDRs, but this merits further investigation.

### Importers for Ferrisiderophores to Enter the Cytoplasm

There are two possible fates for the ferrisiderophore complexes once they are in the periplasm (Figure 2). In most cases, they are directly imported into the cytoplasm. Alternatively, they are subjected to reduction first, and then the released Fe^2+^ molecules are transported into the cytoplasm by ferrous iron transport systems, such as EfeBOU, FeoABC in *E. coli*, FutABC in *Synechocystis* sp. PCC6803 and YfeABCD in *Yersinia pestis* while the free siderophores are exported to the outside the cell for reuse (Schalk and Guillon, 2013; Payne et al., 2015; Lau et al., 2016). In most, if not all, of bacteria in which siderophore transport has been studied, the direct transportation across the IM is the default strategy (Faraldo-Gomez and Sansom, 2003; Schalk and Guillon, 2013). By this strategy, the ferrisiderophore transport is mediated by an ABC transporter, consisting of a siderophore-periplasmic binding protein (PBP) recognizing and binding to the ferrisiderophore, a permease for IM crossing, and an ATPase providing the required energy (Hvorup et al., 2007). Representatives of bacterial ABC transporters for ferrisiderophore import are many, such as *E. coli* FepBC_2_D_2_ (FepB being the PBP, and the dimers FepC_2_ and FepD_2_ forming the permease and the ATPaes, respectively) and FhuDBC_2_ (Shea and McIntosh, 1991; Mademidis and Köster, 1998; Mademidis et al., 2010), *Vibrio anguillarum* FatBC_2_D_2_ and *V. cholerae* VctPDGC_2_ and ViuPDGC_2_ (VctDG and ViuDG, two distinct integral membrane proteins for activity of permease) (Wyckoff et al., 2007; Wyckoff and Payne, 2011). In addition, atypical systems are reported, such as *Y. pestis* YbtPQ, the PBP component is yet unidentified (Perry and Fetherston, 2011). As the *Y. pestis* genome does not encode a homolog of known PBPs, it has been proposed that YbtPQ may not need this component. Furthermore, there are ABC transporters capable of transporting both Fe^3+^ molecules and ferrisiderophore complexes across the IM. A good example is FbpABC, which has been established for 30 years as an Fe^3+^ transporter with FbpA as the Fe^3+^-binding subunit, FbpB as the permease subunit, and FbpC as the ATP-binding subunit in *Serratia marcescens* (Angerer et al., 1990; Braun and Hantke, 2013). Later, multiple studies have demonstrated that this system is able to take up ferrisiderophore complexes in *Neisseria gonorrhoeae* and *B. pertussis* (Strange et al., 2011; Banerjee et al., 2014). In addition, single permeases may also import ferrisiderophore complexes into the cytoplasm (Schalk and Guillon, 2013). Examples include *Sinorhizobium meliloti* RhtX, *P. aeruginosa* FptX and FiuB, *Y. pestis* YbtX, and *L. pneumophila* LbtC (Cuív et al., 2004; Hannauer et al., 2010; Perry and Fetherston, 2011; Chatfield et al., 2012). Although all of these permeases belong to the MFS transporter family, LbtC differs from the former four significantly as it is placed into a different subfamily of MFS (Cianciotto, 2015). Moreover, LbtC shares a greatest level of similarity with FslD/FigD, a protein encoded by a gene in the siderophore operon of *Francisella* (Chatfield et al., 2012).

A large number of ABC transporters, including exporter and importers for fatty acids, macrolide, peptide, hemin and ions are encoded in *S. oneidensis* (Heidelberg et al., 2002). Among them, a FbpABC system (SO_0744-2), by the genome annotation, is the only one that can be confidently linked to Fe^3+^ and ferrisiderophore transport. This system, highly conserved across *Shewanella* species and closely related bacteria in phylogeny (such as *Aeromonas*, *Moritella*, and *Photobacterium*), shows the highest sequence identities to the counterpart of *B. pertussis* (for all three components, E-value < 1e-60) (Table 2). Given that *B. pertussis* FbpABC is required for growth in the presence of native siderophore and xenosiderophores, it is reasonable to propose that this system plays a similar role in *Shewanella*. Importantly, the native siderophore that *B. pertussis* produces is alcaligin, which is structurally similar to all native siderophores found in *Shewanella* (Figure 1). In the case of single-component permeases for ferrisiderophore transport, *S. oneidensis* lacks homologies to the established transporters aforementioned. Despite this, it would be premature to conclude that *Shewanella* do not exploit this mechanism for ferrisiderophore transport as a large portion of single-component permeases remain functionally uncharacterized.

**TABLE 2:** Top five homologs of *S. oneidensis* FbpABC in other bacteria.

Proteins	Homologs	Length (aa)	E-value^a^	Organisms^b^	Annotation
FbpA (335)	FbpA	349	1e-105	*B. pertussis*	Putative iron binding protein
	FutA1	360	1.1E-80	*Synechocystis sp.*	Iron uptake protein A1
	PA5217	332	3.2E-73	*P. aeruginosa*	Probable binding protein component of ABC iron transporter
	FutA2	346	1.7E-70	*Synechocystis sp.*	Iron uptake protein A2
	Fbp	330	9.3E-24	*N. gonorrhoeae*	Major ferric iron-binding protein
FbpB (544)	FbpB	556	6.0E-107	*B. pertussis*	Ferric iron ABC transporter, permease protein
	HI_0098	506	4.8E-38	*H. influenzae*	Fe^3+^-transport system permease protein FbpB 2
	P21409	527	7.5E-29	*S. marcescens*	Fe^3+^-transport system permease protein SfuB
	FbpB1	632	1.8E-16	*H. influenzae*	Fe^3+^-transport system permease protein FbpB 1
	FbpB	687	4.5E-16	*A.pleuropneumoniae*	Fe^3+^-transport system permease protein FbpB
FbpC (349)	FbpC	348	2.9E-73	*E. coli*	Fe^3+^ import ATP-binding protein FbpC
	FbpC	360	1.4E-70	*M. japonicum*	Fe^3+^ import ATP-binding protein FbpC
	FbpC	353	2.4E-70	*B. abortus*	Fe^3+^ import ATP-binding protein FbpC
	FbpC	354	2.4E-70	*P. fluorescens*	Fe^3+^ import ATP-binding protein FbpC
	FbpC	354	6.0E-67	*B. pertussis*	ABC transport protein, ATP-binding component

*^a^BLASTp E-value.*

*^b^Genus names from the top: B, Bordetella; P, Pseudomonas; N, Neisseria; H, Haemophilus; S, Serratia; A, Actinobacillus; E, Escherichia; M, Mesorhizobium; B, Brucella.*

### Ferrisiderophore Dissociation

Given the extremely high affinity of siderophores for ferric iron, ferrisiderophore complexes are thermodynamically very stable, causing ferrisiderophore dissociation a challenge to the cell. Although studies into bacterial ferrisiderophore dissociation are still rather limited, several mechanisms for iron release from the chelated siderophores have been postulated, associated either with a siderophore hydrolysis, chemical modification of the siderophore such as acetylation, or proton-mediated iron release (Schalk and Guillon, 2013; Table 3). Given that the stability constants for ferrisiderophores (∼30) are substantially larger than those (less than 10) for ferrous-siderophore and the siderophore complexes formed with other metals, iron-releasing via reduction of the coordinated ferric iron is regarded to be vital because all of the mechanisms eventually lead to iron reduction (Evers et al., 1989; Hernlem et al., 1996; Holt et al., 2005; Miethke and Marahiel, 2007; Hannauer et al., 2010; Miethke et al., 2011). In most of studied examples, ferrisiderophore dissociation occurs in the cytoplasm (Schalk and Guillon, 2013; Figure 2). A common strategy for ferrisiderophore dissociation is first to modify the siderophore scaffold. Fes of *E. coli*, a cytoplasmic esterase, is the first known to perform siderophore hydrolysis for iron releasing (Brickman and McIntosh, 1992). Subsequently, equivalent enzymes have been identified in other bacteria, such as IroD and IroE found in *E. coli* and *Salmonella*, *B. subtilis* BesA, and *Bacillus halodurans* FchR (Lin et al., 2005; Zhu et al., 2005; Miethke et al., 2006, 2011; Abergel et al., 2009; Perraud et al., 2018).

**TABLE 3:** Dissociation mechanism of ferrisiderophore.

Ferrisiderophore	Dissociation mechanism	Kinetics data		Ref.
		Enzyme	Catalytic efficiency (*k*_cat_/*K*_m_)(μM^–1^min^–1^)	
Fe^3+^-enterobactin	Hydrolyzed by esterase then metal reduction with thiol or other reductase	Fes	>90	Lin et al., 2005
		IroD	617	
		IroE	0.9	
Ferribacillin	Hydrolyzed by esterase then metal reduction with other reductase	BesA	380	Miethke and Marahiel, 2007
Fe^3+^-enterobactin	Reduced in the presence of NADPH as the preferred electron donor substrate	YqjH (ViuB)	0.49	Miethke et al., 2011
Fe^3+^-vibriobactin			0.076	
Fe^3+^-Aerobactin			0.0012	
Ferrichrome	Iron is released from ferrichrome in the cytoplasm by a mechanism involving iron reduction followed by acetylation of the desferrisiderophore	FhuF	–	Hannauer et al., 2010; Matzanke et al., 2004

In the bacterial cytoplasm, ferrisiderophore reduction involves the superfamily of siderophore-interacting proteins (SIP) composed of two distinct families: FSR family and SIP family (Cain and Smith, 2021). The best understood FSR is *E. coli* FhuF, which uses a [2Fe-2S] cluster and catalyzes reduction of iron in the ferrisiderophore (desferrichrome and ferrioxamine) (Matzanke et al., 2004). Although the FhuF homologs are not widely distributed, they are found in *P. aeruginosa* and a couple of *Rhizobium* species (Capela et al., 2001; Llamas et al., 2006). Additionally, FSRs with homology to a family of NAD(P)H:flavin oxidoreductases have been identified. In *Thermobifida fusca*, FscN uses a bound FAD cofactor and NADH to reduce the mixed catecholate-hydroxamate siderophore (Li K. et al., 2016). Moreover, NADPH-dependent flavoproteins have been found to combine both siderophore-degrading and reducing activities. *E. coli* YqjH, which is wide-spread among bacteria, is not only a reductase but also an efficient hydrolyase for ferrienterobactin (Miethke et al., 2011). In a rare case, the reduction in the cytoplasm can be performed by a functional domain of a large membrane-spanning protein, IrtAB of *Mycobacterium tuberculosis*, which is an ABC transporter for the import of ferric-siderophore (carboxymycobactin) (Arnold et al., 2020).

Ferrisiderophore dissociation could occur in the periplasm too (Figure 2). In *P. aeruginosa*, the ferrisiderophore (pyoverdine) in the periplasm is caught by FpvC and FpvF, the PBPs of ABC transporter FpvCFD_2_E_2_ (Brillet et al., 2012). Reduction is carried out by FSR FpvG on the IM containing a total of 4 TM α helices (Ganne et al., 2017), resulting in iron releasing, and then released iron is imported into the cytoplasm by FpvDE while the apo-siderophore is recycled into extracellular environments for reuse (Bonneau et al., 2020). *P. aeruginosa* also possesses another IM-anchored FSR, FoxB, which is encoded by a gene immediately downstream of a TBSR gene within the same operon (Josts et al., 2021). This enzyme is a di-heme protein consisting of a four TM helical bundle capped by two PepSY (peptidase propeptide and YpeB domain) domains. In contrast, hemoprotein FrcB of α-proteobacterium *Bradyrhizobium japonicum*, is shown to be a stand-alone FSR (Small and O’Brian, 2011). Despite the lack of sequence similarity, FrcB has a four TM helical bundle that resembles FpvG and FoxB, implying that the topology is conserved in these membrane-bound FSRs. Moreover, in the periplasm enzymes that carry out iron-releasing by modify the siderophore scaffold have also been found, such as Cee of *Campylobacter* (Zeng et al., 2013).

In *Shewanella*, genes for siderophore biosynthesis are organized into a single operon constituting either *pubABC* as in *S. oneidensis*, or *avbABCD* as in *S. algae* B516 (Wang et al., 2020; Figure 3A). In both cases, the *putB* gene is always in the proximity of the operon encoding either PubABC- or AvbBCD-type siderophore synthetic system (Wang et al., 2020; Figure 3A). Although the genome annotation and sequence analyses suggest that PutB is an FSR in the cytoplasm, its physiological role could not be directly verified with a single-gene knockout. This is because the absence of PutA causes a severe defect in cytochrome *c* biosynthesis (will be discussed below), that is not observed from the *putB* mutant (Dong et al., 2017). Despite this, the essentiality of PutB for iron uptake mediated by siderophores produced endogenously is validated in strains in which the Feo system is additionally removed. The rational is that without the Feo system, the siderophore-mediated iron-uptake becomes nearly essential for growth of *S. oneidensis*. The observation that the *putA feo* and *putB feo* double mutants are indistinguishable from each other in growth supports that PutB is required for ferrisiderophore dissociation (Liu et al., 2018).

**FIGURE 4:**
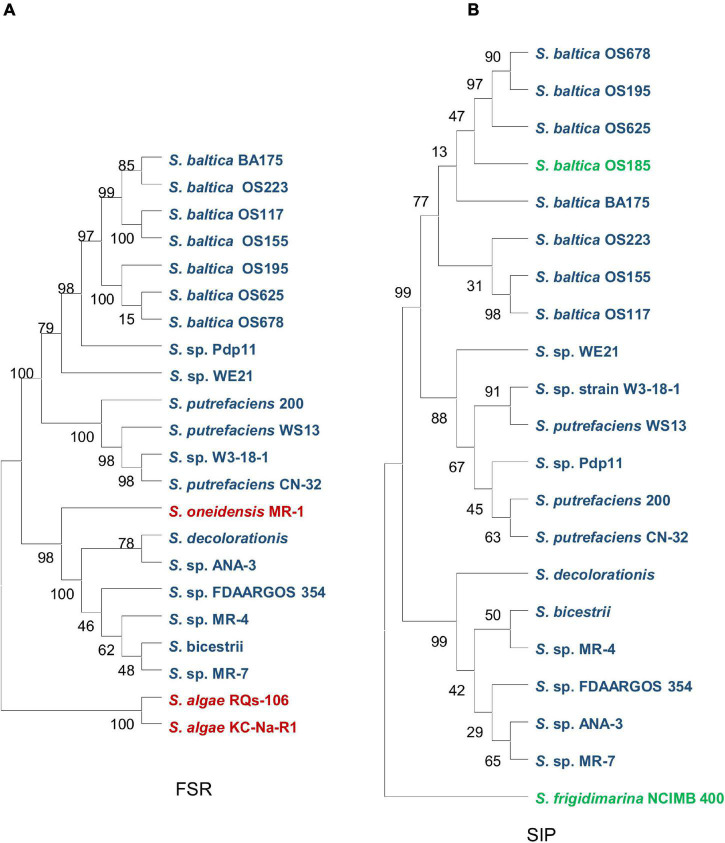
Phylogenetic analysis of two ferricsiderophore reduction families in *Shewanella* spp. **(A)** The ferric-siderophore reductase (FSR) family. **(B)** The siderophore-interacting protein (SIP) family. Strains containing only FSR, only SIP, and both are in red, green, and blue, respectively.

Siderophore-interacting proteins have also been identified in *Shewanella* (Trindade et al., 2019). The SIP (SFRI_RS12295) of *Shewanella frigidimarina*, whose structure is available, is able to bind and reduce hydroxamates, including all siderophores produced by *Shewanella* species. This SIP uses either ferredoxin or NAD(P)H as an electron donor for reduction, although the use of NAD(P)H requires the presence of an Fe^2+^-chelating agent (Trindade et al., 2019). Interestingly, some *Shewanella* species contain SIPs only, such as *S. frigidimarina*, some have FSRs only like *S. oneidensis* and *S. algae*, and a few species host both of them, like *S. putrefaciens* and *Shewanella baltica* (Trindade et al., 2019; Figure 4).

## Regulation of Siderophore Biosynthesis

Most of microbes are unable to survive without iron, but the overloaded intracellular iron is toxic to cells by causing biomolecular damages to DNA, proteins and lipids via Fenton reaction (Imlay, 2013). Iron homeostasis, therefore, which requires coordination between iron acquisition and consumption, including iron uptake, consumption, storage, and efflux, must be carefully maintained (Andrews et al., 2013). In many bacteria, transcription factor Fur plays a primary role to orchestrate the coordination by sensing intracellular iron levels and regulating transcription of genes involved in iron acquisition and consumption (Troxell and Hassan, 2013). Recently, there have been reports suggesting that Fur as a global regulator modulates transcription of genes implicated in diverse biological processes via complex and varying mechanisms depending on the microbes (Fillat, 2014; Seo et al., 2014; Roncarati et al., 2016). Moreover, Fur is also capable of interacting with a [2Fe-2S] cluster to sense intracellular iron homeostasis (Fontenot et al., 2020).

Despite these, the classical Fur regulation still lies at the center (Fleischhacker and Kiley, 2011; Butcher et al., 2012; Fillat, 2014; Pi and Helmann, 2017). In this paradigm, Fur binds to Fe^2+^ and the dimeric Fe^2+^–Fur complex (holo-Fur) recognizes target sequences (Fur-box) upstream of iron-regulated genes and represses their transcription under iron-replete conditions and de-repression occurs under iron-limiting conditions (Fillat, 2014). One of these genes encodes small RNA RyhB, which acts posttranscriptionally to promote an iron-sparing response by downregulating the translation of proteins with iron cofactors, preserving iron for essential proteins (Massé and Gottesman, 2002; Chareyre and Mandin, 2018; Banerjee et al., 2020). Fur proteins act as a homodimer of polypeptides consisting of an N-terminal DNA-binding domain linked by a hinge region to a C-terminal dimerization domain (Butcher et al., 2012). Several Fur-box variations ranging from 15 to 23 bp have been reported (19-bp in *E. coli*), but all of them are characterized to be AT rich, largely built on the base sequence GATAAT, and share considerable sequence similarities (Baichoo et al., 2002; Fillat, 2014). In addition, a few two-component systems (TCSs) have been demonstrated to be involved in regulation of siderophore biosynthesis, for instance, BarA/UvrY (sensor kinase/response regulator) (Zhang and Normark, 1996; Pernestig et al., 2002; Frangipani et al., 2014).

Many bacterial genes involved in siderophore biology have been identified to be under the direct control of Fur and RyhB, especially since omics technology emerged (Seo et al., 2014; Beauchene et al., 2015; Chareyre and Mandin, 2018; Banerjee et al., 2020). Taking *E. coli* as example, the biosynthesis of siderophore aerobactin operon *iucABCD* is repressed by Fur (De et al., 1987). Similarly, a *fur* mutant produces more siderophores, including enterbactin, salmochelin, and aerobactin, and at least the enterobactin gene cluster (6 operons, including *ent* for biosynthesis and *fep* for transporters) is under Fur regulation (Crosa and Walsh, 2002). Additional mechanisms involve the overexpression of RyhB in *fur* mutants, leading to activation of the expression of *shiA* (encoding transporter of shikimate, a precursor of enterbactin and salmochelin biosynthesis) and *cirA* (encoding a TBDR for certain ferrisiderophores) (Prévost et al., 2007; Salvail et al., 2013; Porcheron et al., 2014).

*Shewanella oneidensis* possesses both Fur and RyhB (Wan et al., 2004; Yang et al., 2010). The Fur loss lowers concentrations of the total iron but increases free iron, a phenomenon attributed to constitutive and repressive expression of iron transport and iron usage proteins respectively (Fu et al., 2018; Liu et al., 2020). The Fur box of *S. oneidensis* is similar to that of *E. coli* and the members of the predicted Fur regulons include operons for TBDRs *irgA* and SO_1482, siderophore biosynthesis enzyme *pub*, and ferrous transporter *feo* (Yang et al., 2009; Fu et al., 2018). However, in contrast to *E. coli*, *S. oneidensis* strains lacking Fur, which is no longer responsive to changes in exogenous iron levels, have lowered siderophore production (Fu et al., 2018). This unexpected phenomenon may be linked to RyhB as its physiological impacts appear to differ from those reported in *E. coli* profoundly (Yang et al., 2010; Meibom et al., 2018). Nonetheless, to date there has not been any evidence associating RyhB with the biosynthesis and transport of siderophores in *S. oneidensis*.

In *S. oneidensis*, an orphan response regulator of TCS, SO_2426, is essential for siderophore biosynthesis whereas the involvement of BarA/UvrY in siderophore biology remains unknown (Binnenkade et al., 2011; Henne et al., 2011). Expression of SO_2426 is transcriptionally responsive to acid and heavy metal stresses and is upregulated in the *fur* mutant (Chourey et al., 2008; Yang et al., 2008, 2009). SO_2426 interacts with the promoter region of the *pub* operon to activate transcription (Henne et al., 2011). Thus, molecular mechanisms controlling iron homeostasis in *S. oneidensis* likely rely on complex regulation by Fur, a combination of both direct and hierarchical effect. First, Fur mediates transcriptional repression of its target genes, which includes those for iron uptake, storage and consumption as well as for regulators RyhB and SO_2426. Second, when produced at altered levels, RyhB and SO_2426 exert their physiological impacts by either counteracting or enhancing the influence of Fur regulation. It should be noted that several of the operons predicted to be the SO_2426 regulon members also have Fur box in their upstream regions, suggesting that their expression is likely a result of coordinated or antagonistic effects of Fur and SO_2426 (Henne et al., 2011).

## Unusual Physiological Impacts of Siderophores

Siderophores confer microbes a practical and efficient strategy to acquire iron for survival and fitness gain in natural environments, especially under oxic conditions. Consequently, impacts of siderophores on bacterial physiology have been found to be diverse and profound, far beyond simple iron chelation (Wilson et al., 2016; Kramer et al., 2020). Biological processes in which siderophores have been demonstrated to be involved include transport of non-iron metals, sequestration of toxic metals, regulation as signals, protection from oxidative stress, modulation of antibiotic activity. Additionally, siderophores have recently been regarded to play an important role in mediating social interactions between individuals in biological communities, and between members of microbial assemblies and the eukaryotic hosts that they inhabit (Kramer et al., 2020).

**FIGURE 5:**
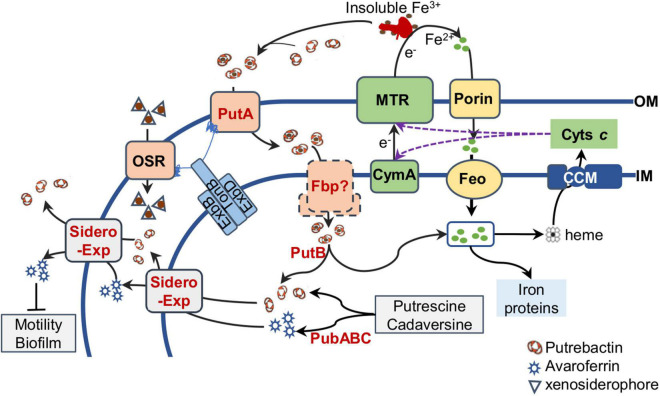
Siderophore biology in *S. oneidensis*. Siderophores (putrebactin and avaroferrin) are synthesized by PubABC in the cytoplasm and exported by unknown exporters (Sidero Exp) on the IM (inner membrane) and OM (outer membrane). By binding to Fe^3+^ of insoluble Fe^3+^ compounds, siderophores promote solubility of insoluble Fe^3+^ compunds and form ferrisiderophores, which are imported into the periplasm by TBDR PutA and then probably transported across the IM by Fbp ATP transporter. Ferrisiderophores are reduced by FSR PutB, resulting in Fe^2+^ release. Fe^2+^ can also be imported into the cell through porins on the OM and FeoAB system on the IM. The intracellular Fe^2+^ pool is maintained in balance to ensure biosynthesis of iron proteins and heme, which is the cofactor for cytochromes *c* synthesized by the cytochrome *c* maturation (CCM) system on the IM. Matured cytochromes *c*, including CymA and the metal reducing (MTR) system that convert Fe^3+^ to Fe^2+^ extracellularly with electrons from the quinol pool in the IM (not shown), confer *S. oneidensis* cells respiratory versatility. OSR, other siderophore receptor, important for import of iron-xenosiderophores released by other bacteria. Avaroferrin has additional activity affecting motility and biofilm.

*Shewanella* are exceptionally abundant in cytochromes *c*, iron-based proteins serving as the foundation for their renowned respiratory versatility. In line with this feature, *S. oneidensis* has an iron content substantially higher than *E. coli*, by approximately 4 fold (Daly et al., 2004). However, this comes at cost. Compared to both *E. coli* and radiation-resistant *Deinococcus radiodurans*, *S. oneidensis* is highly sensitive to ROS, whose killing effect is greatly boosted by Fe^2+^ (Jiang et al., 2014; Shi et al., 2015; Wan et al., 2018). Naturally, iron homeostasis is particularly important for this group of bacteria to survive and thrive in their native environments. As illustrated in Figure 5, the Feo system and the siderophore-based system of *S. oneidensis* constitute predominant routes for iron uptake although there is a secondary ferrous iron importer encoded in the genome (Bennett et al., 2018; Liu et al., 2018). *S. oneidensis* employs Feo as the main iron acquisition system, whose absence results in a substantial growth defect, contrasting that no significant influence on growth is observed from the loss of siderophore synthetase (the *pub* mutant) (Liu et al., 2018). Moreover, even under aerobic conditions, iron reduction occurs in *S. oneidensis*, a reaction that provides Fe^2+^ for the Feo system (Yuan et al., 2013). Clearly, as long as cells are capable of carrying out iron reduction, iron can be imported via Feo when it is in place. Interestingly, the cytochrome *c* content is not affected significantly by the depletion of the Feo system, suggesting that iron homeostasis is maintained properly despite the vast differences in growth rates. This observation has an important implication, that is, cells are able to adjust growth rates in accord with the iron-uptake capacity in order to sufficiently supports the respiratory versatility.

In contrast to the loss of siderophore synthetase, the absence of PutA, the TBDR specific for endogenous siderophores, causes a significant reduction in the cytochrome *c* content (Dong et al., 2017; Liu et al., 2018). This effect resembles that resulting from the addition of excessive iron chelators, such as 2,2-dipyridyl and siderophores that *S. oneidensis* cells could not import, such as desferrioxamine (Liu et al., 2018). The underlying mechanism is that the *putA* mutant synthesizes siderophores constitutively, and after released to the outside of the cell, these siderophores, the same as exogenous iron chelators, bind to Fe^3+^ in the environments. As a result, the concentrations of extracellular iron become too low for iron ions to be imported (Liu et al., 2018).

To date, there have been some reports about physiological influences of siderophores produced by *Shewanella* endogenously. Most of them are attributed to the altered iron availability associated with siderophores. It was demonstrated more than 15 years ago that Fe^3+^-respiring *S. putrefaciens* strain 200 entails Fe^3+^ destabilization by producing organic ligands with high Fe^3+^-chelating capability, presumably putrebactin (Taillefert et al., 2007; Figure 5). Although the identity of these organic ligands was not revealed then, it is highly possible that they are siderophores because putrebactin plays a critical role in Fe^3+^ solubilization during aerobic respiration (Fennessey et al., 2010). Siderophores synthesized by PubABC have also been shown to be important for manganese-oxide reduction (Kouzuma et al., 2012). The mechanism underpinning this phenomenon is proposed to be substantial reduction in the contents of total iron and cytochromes *c*. However, this is at odds with the findings of more recent studies that the absence of PubABC only marginally impacts the cellular concentrations of the both (Dong et al., 2017; Liu et al., 2018, 2020).

In *Shewanella*, endogenous siderophores have also been demonstrated to be involved in biological processes other than those directly related to iron availability (Figure 5). A study reveals that the disruption of *speF*, an essential gene for biosynthesis of putrescine, improves biofilm cohesiveness and performance in Cr^4+^ immobilization in *S. oneidensis* (Ding et al., 2014). Given that putrescine is one of the common polyamines and polyamines play a critical role in many biological processes, including binding to nucleic acids, stabilizing outer membranes, and protecting cells from toxic effects of ROS and acid stresses, and biofilm formation in bacteria (Patel et al., 2006; McGinnis et al., 2009; Michael, 2016), the phenotype caused by the *speF* disruption is linked to polyamines. However, the possibility that siderophores are involved could not be ruled out because putrescine is the most important and abundant substrate for siderophores produced endogenously in *S. oneidensis* (Kadi et al., 2008a; Soe et al., 2012; Rütschlin et al., 2018; Wang et al., 2020). More importantly, SpeF dictates biosynthesis of cadaverine while putrescine biosynthesis is primarily, if not exclusively, catalyzed by the ADC (SpeA-Agu) pathway in *S. oneidensis* (Wang et al., 2020). Therefore, it is tempting to speculate that the absence of cadaverine, and/or its derivatives may be responsible for the enhanced biofilm formation (Ding et al., 2014). Coincidently, avaroferrin, a siderophore synthesized from a combination of putrescine and cadaverine by *S. algae* B516, inhibits swarming of *Vibrio alginolyticus* (Böttcher and Clardy, 2014). Given that the motility and biofilm formation are two closely interplaying processes (Jenal et al., 2017), it is conceivable that avaroferrin may also regulate biofilm formation in *S. oneidensis*.

## Conclusion

The biosynthesis, physiological impacts, and application of siderophores have been of interest and intensive studies have been carried out in *E. coli* and other model bacteria, pathogens in particular, since their discovery about 80 years ago. As a consequence, our current understanding of bacterial siderophore biology has been unprecedentedly profound. Nevertheless, one inevitable shortcoming would be that the current research on the subject is biased toward a few model bacteria and particular siderophores. As bacteria represent the most diverse group of living organisms on the Earth and environmental circumstances that bacteria face in their daily life are so ever-changing, it is certain that distinct synthetic systems and mechanisms may evolve to generate varying siderophores and regulate their activity to cope with iron scarcity.

To elucidate the siderophore biology in *Shewanella*, facultative anaerobes with a huge demand of iron, this review provides new insights into an incredibly complex network governing how these bacteria synthesize, transport, and utilize siderophores, and the regulation of these processes in response to iron shortage. Nevertheless, some basic questions remain under the veil. What are exporters and importers for siderophore and ferrisiderophore respectively? What are substrates of TBDTs other than PutA? What is the sensor kinase for SO_2426 and what is the signal that it perceives in *S. oneidensis*? More importantly, how are siderophores involved in intra- and interspecies cooperation and competition, and thus facilitate the bacteria to thrive in the redox stratified niches? In the light of all these unanswered questions, we believe that the understanding of the siderophore biology in *Shewanella* that we have had to date is rather limited, and there is still much to be discovered. In addition, how siderophores, especially those produced by *Shewanella*, could be engineered to contribute in drug production and therapy remains to be explored.

## Author Contributions

All authors listed have made a substantial, direct, and intellectual contribution to the work, and approved it for publication.

## Conflict of Interest

The authors declare that the research was conducted in the absence of any commercial or financial relationships that could be construed as a potential conflict of interest.

## Publisher’s Note

All claims expressed in this article are solely those of the authors and do not necessarily represent those of their affiliated organizations, or those of the publisher, the editors and the reviewers. Any product that may be evaluated in this article, or claim that may be made by its manufacturer, is not guaranteed or endorsed by the publisher.

## Abbreviations

ABC: ATP-binding cassette
ADC: arginine decarboxylase
BAAD: basic amino acid decarboxylases
FSR: ferrisiderophore reductase
Fur: ferric uptake regulator
IM: inner-membrane
LDC: lysine decarboxylase
MFS: major facilitator superfamily
NIS: NRPS-independent
NRPS: non-ribosomal peptide synthetase
ODC: ornithine decarboxylase
OM: outer-membrane
PBP: periplasmic binding protein
RND: resistance-nodulation-division
ROS: reactive oxygen species
SIP: siderophore-interacting protein
TBDR: TonB-dependent receptor
TCS: two-component system
TM: transmembrane.
